# Sandwich Plate Structure Periodically Attached by S-Shaped Oscillators for Low Frequency Ship Vibration Isolation

**DOI:** 10.3390/ma16062467

**Published:** 2023-03-20

**Authors:** Chaoming Shen, Jie Huang, Zexin Zhang, Jingya Xue, Denghui Qian

**Affiliations:** 1 School of Naval Architecture & Ocean Engineering, Jiangsu University of Science and Technology, Zhenjiang 212100, China; 2 School of Civil Engineering & Architecture, Jiangsu University of Science and Technology, Zhenjiang 212100, China

**Keywords:** locally resonant phononic crystal, band gap characteristic, band structure, flexural wave, ship vibration isolation

## Abstract

Locally resonant phononic crystals are a kind of artificial periodic composite material/structure with an elastic wave band gap that show attractive application potential in low-frequency vibration control. For low-frequency vibration control problems of ship power systems, this paper proposes a phononic crystal board structure, and based on the Bloch theorem of periodic structure, it uses a finite element method to calculate the band structure and the displacement fields corresponding to the characteristic mode and vibration transmission curve of the corresponding finite periodic sandwich plate structure, and the band gap characteristics are studied. The mechanism of band gap formation is mainly attributed to the mode coupling of the phononic crystal plate structure. Numerical results show that the sandwich plate structure has a double periodicity, so it has a multi-stage elastic wave band gap, which can fully inhibit the transmission of flexural waves and isolate the low-frequency flexural vibration. The experimental measurements of flexural vibration transmission spectra were conducted to validate the accuracy and reliability of the numerical calculation method. On this basis, the potential application of the proposed vibration isolation method in a marine power system is discussed. A vibration isolation platform mounted on a steel plate is studied by numerical simulation, which can isolate low-frequency vibration to protect electronic equipment and precision instruments.

## 1. Introduction

With the rapid development of the shipping industry, people pay more and more attention to the safety and comfort of shipping. Ship vibration and noise not only affect the physical and mental health of people but also cause fatigue damage and shorten the service lives of instruments. Since a ship is a complex structure, its vibration sources are very complex and vary. On the one hand, the ship is subjected to external forces such as wind load and wave load; on the other hand, the vibration of mechanical equipment in the ship, such as the vibration of the main engine and torsional vibration of the propeller, causes the coupling of elastic waves to be very complicated. Traditional measures to reduce vibration mainly include suppressing the vibration source’s intensity, cutting off the propagation path, and providing receiver protection, among which the most commonly used is to start from the propagation path to solve the vibration problem. Plate structure is one of the basic components and the main propagation path, and it has been one of the main research objects in the field of vibration control for many years. Solving the vibration reduction problem of plate structures means solving many problems of vibration reduction in marine engineering. The vibration band gap characteristics of phononic crystals make the propagation of vibration within the band gap prohibited or suppressed to a large extent, which provides a new idea for its application in the field of vibration reduction.

Phononic crystal [1] is an artificial periodic composite material/structure with an elastic wave band gap that has attracted extensive interest from scholars at home and abroad in recent years. Due to the elastic band gap characteristics [2,3,4,5] of the phononic crystal structure, vibration will be difficult to propagate in the corresponding band gap, so there are many potential applications in vibration and noise reduction [6,7,8,9,10,11,12,13,14] that can be widely used in ships, aircraft, and other engineering fields. In existing studies, Bragg scattering [15] and local resonance [16] are the main mechanisms of band gap formation, between which locally resonant phononic crystals can generate low-frequency band gaps. The results show that a phononic crystal plate structure [17,18,19,20,21] also has band gap characteristics. Qian et al. [17] designed a two-layer plate structure of locally resonant phononic crystals that can open a complete band gap with a low initial frequency and a wide band gap. Li et al. [19] studied low-frequency and broadband elastic wave attenuation and vibration suppression of plate-type elastic metamaterials. Miranda et al. [20] studied the complex band structure of metamaterial plates with spring-mass resonators by using the plane-wave expansion method. Song et al. [21] calculated the band structure and transmission characteristics of sandwich metamaterials and bare sandwich plates, respectively, to study the influence of periodic design on vibration and sound radiation.

Therefore, by introducing the design idea of phononic crystals into some basic structures, the local resonant phononic crystal sandwich plate formed by this paper provides a new idea for low-frequency vibration and noise control, which is difficult to realize in the shipping field. At present, many people have developed useful achievements in the application of phononic crystals in the field of ship vibration isolation. Song et al. [6] studied the suppression of vibration and noise radiation in a flexible raft system by using the periodic structure theory and calculated the dynamic response of the system and the dispersion relationship of the periodic structure. Li et al. [22] studied the flexural band gap and vibration attenuation characteristics of marine periodic bidirectional orthogonal reinforced plates. However, the band gap of the flexural wave of the periodic bidirectional orthogonal reinforcement plate is mainly concentrated in the middle and high frequencies, which makes it difficult to solve the problem of low-frequency vibration isolation of ships. Ruan et al. [23] designed and analyzed a single-phase helical locally resonant phononic crystal for vertical vibration isolation of a certain type of ship. Numerical results and experiments show that the structure can isolate low-frequency vibration. However, with the introduction of helical phononic crystals, it is difficult to guarantee the strength and stiffness of ship structures. Chen et al. [24] proposed a ship vibration isolation method using the band gap concept of a sandwich plate elastic element structure to solve the low-frequency vibration and noise control problems of marine power systems and discussed the potential application of the proposed sandwich plate elastic element structure in vibration control of marine power systems.

Based on the existing phononic crystal structure research, the author first designs the locally resonant oscillator, calculates the band structure, and obtains the elastic wave band gap at medium and low frequencies. Then three vibrators were arranged in vertical periodicity, and the top and bottom plates were attached to form new crystal cells. Then the phononic crystal plate structure is constructed by arranging the new cell period. The finite element method is used to calculate the band structure, and the low-frequency band gap of 56–97 Hz and the middle-frequency band gap of 251–647 Hz are obtained, which can solve the low-frequency vibration problem of ships to a certain extent. The experimental measurements of the acceleration frequency responses were conducted to validate the accuracy and reliability of the numerical calculation method. Finally, the potential application of the proposed sandwich plate structure in marine power system vibration control is discussed.

## 2. Model and Method

**Figure 1:**
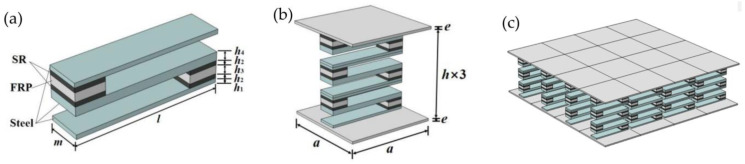
(**a**) S-type oscillator. (**b**) Phononic crystal plate cell structure. (**c**) Phononic crystal plate structure.

According to the band gap formation mechanism of the locally resonant phononic crystal structure, the S-type oscillator is designed, and its schematic diagram is shown in Figure 1a. As can be seen from the figure, Figure 1a is composed of three steel plates and two composite blocks interlaced with each other. The upper and lower steel plates are thin plates with identical dimensions, while the middle steel plate is a thick plate with thicker dimensions, and the composite blocks are composed of rubber-fiber reinforced plastics (FRP)-rubber. Eight S-shaped oscillators were arranged vertically and periodically as interlayers, and the top and bottom plates, composed of fiber-reinforced plastics, were attached above and below them to form crystal cells of a phononic crystal plate structure, as shown in Figure 1b. The crystal cells are arranged periodically to form the phononic crystal plate structure, as shown in Figure 1c. In the structural model, the lattice constant, the thickness of top and bottom plates, the S-type oscillator thin plate, the rubber layer, the FRP layer, the S-type oscillator thick plate, and the total height of the oscillator are *a*, *e*, *h*_1_, *h*_2_, *h*_3_, *h*_4_, and *h*, respectively. As a unit of the oscillator, the length and width of the S-type oscillator are l and m, respectively, as shown in Figure 1. In the structural model, the geometric parameters are shown in Table 1 and Table 2. The material parameters of FRP are: density m = 1800 kg/m^3^; Young’s modulus E_1_ = E_2_ = 23.4 GPa, E_3_ = 13.5 GPa; Poisson’s ratio v_1_ = v_2_ = 0.22, v_3_ = 0.13.

For the band structure of a single S-type oscillator, the finite element method is used to calculate it with the help of the commercial software COMSOL Mutiphysics. A tetrahedral mesh is used to divide the finite element mesh. For the boundary conditions of S-type oscillators, the non-contact surfaces are in the condition of a free boundary without pressure. The periodic boundary conditions derived from the Bloch–Floquet theorem [25] are adopted for the adjacent cell interface:

(1) $$u_{i}(z+a)=u_{i}(z)e^{-i(k_{z}a)}$$

where *u_i_* denotes the elastic displacement along the *x*, *y*, and *z* directions, respectively, when i is equal to *x*, *y*, and *z*; *k_z_* is the components of the Bloch wave vector limited in the irreducible first Brillouin zone (1BZ). Substituting the periodic boundary condition from Equation (1) into the finite element characteristic equation of free vibration, the result can be obtained.

***K**u* = *ω*^2^***M**u* (2)

In Equation (2), both the stiffness matrix ***K*** and the mass matrix ***M*** contain the terms of the Bloch wave vector coupling, which are different from the stiffness matrix and mass matrix in the traditional finite element characteristic equation. Equation (2) is a typical generalized eigenvalue problem for ω^2^. For each given Bloch wave vector, a corresponding series of eigenfrequencies can be obtained by solving the eigenvalues. By traversing all the wave vectors on the boundary of the irreducible Brillouin region, the band structure of the cell can be obtained. A wide band gap is opened in the band structure diagram, so it is of theoretical significance to deal with the problem of vibration and noise reduction in engineering in the frequency domain of this band gap. The S-type vibrators were placed on the top and bottom plates to form new crystal cells, and then the new crystal cells were arranged in a periodic manner so as to construct the sandwich plate structure of phononic crystals that can handle vibration and noise reduction in engineering, as shown in Figure 1c.

The period number of the S-type oscillator also has an important influence on the band gap. Theoretically, the more cycles, the better the vibration isolation effect. Actually, due to the restrictions on the size of the structure, lightweight requirements, etc., it is necessary to use a small period to meet the desired damping effect. For the phononic crystal plate structure, at least two oscillators are required to make better use of the band gap characteristics of the S-type oscillators and achieve the vibration-damping effect. In this paper, a sandwich plate structure with a 3-period S-type oscillator is taken as an example. Three S-type oscillators are arranged vertically and periodically, and the upper plate and lower plate are attached above and below them to form the cell of a phononic crystal plate structure. The schematic diagram of the structure is shown in Figure 1b. The finite element method is also used to calculate the band structure of the phononic crystal plate cell. As for the boundary conditions, the non-contact surfaces all adopt the free boundary conditions without pressure, and the periodic boundary conditions derived from the Bloch–Floquet theorem are used for the adjacent cell interface:

(3) $$u_{i}(x+a,y+a)=u_{i}(x,y)e^{-i(k_{x}a+k_{y}a)}$$

where *u_i_* denotes the elastic displacement along the *x*, *y*, and *z* directions, respectively, when i is equal to *x*, *y*, and *z*; *k_x_* and *k_y_* are components of the Bloch wave vector limited in the irreducible first Brillouin zone (1BZ). Substituting the periodic boundary condition from Equation (3) into the finite element characteristic equation of free vibration. The band structure of the crystal cell can be obtained in the same way as that of the S-type oscillator.

## 3. Results and Analyses

### 3.1. Band Structure, Eigenmode, and Transmission Power Spectrum of the S-Type Oscillator

**Figure 2:**
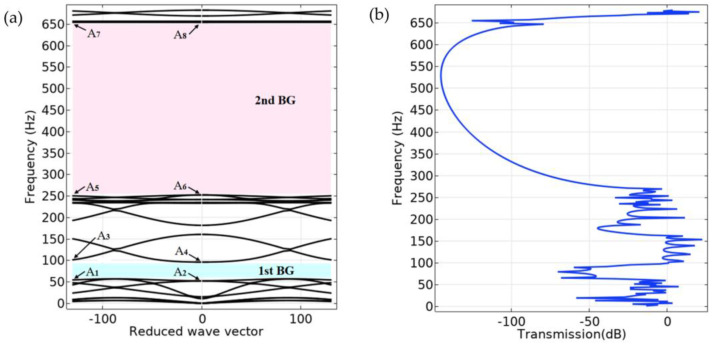
(**a**) Band structure of the proposed S-type oscillator. (**b**) Transmission power spectrum of S-type oscillator with three periods.

**Figure 3:**
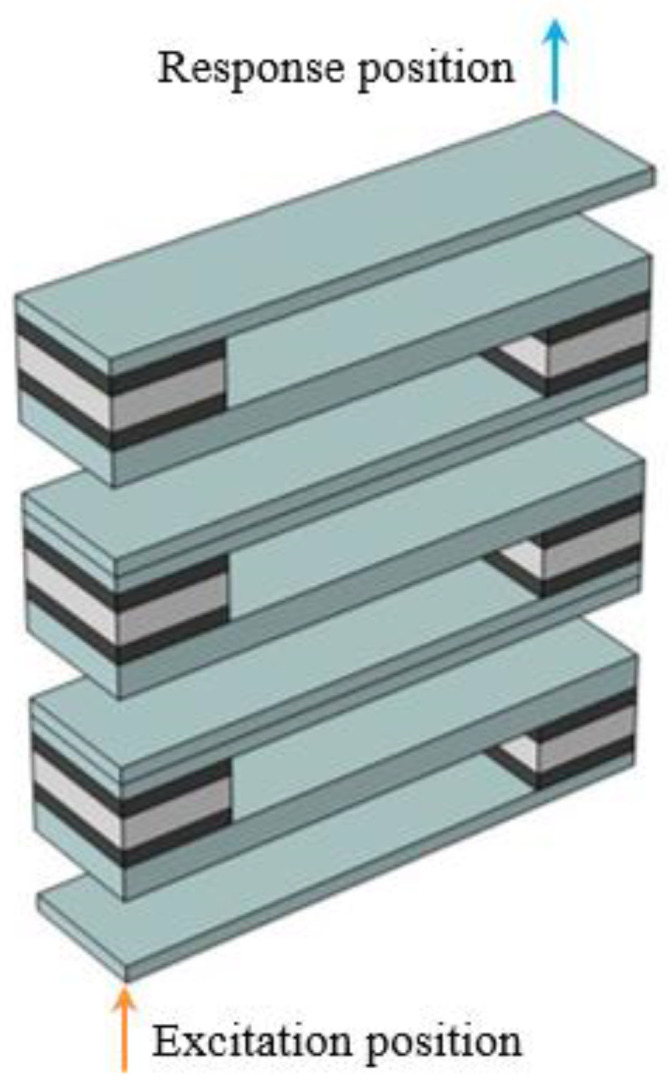
The oscillator structure model is formed by the periodic arrangement of 3 S-shaped unit cells.

Figure 2a shows the energy band structure of the S-type oscillator, and the material parameters and geometric parameters used in the calculation are shown in Table 1 and Table 2, respectively. In order to verify the accuracy of the calculated band structure, three S-shaped oscillators are arranged vertically periodically, and the excitation position and response position are placed at the bottom and top of them, respectively, as shown in Figure 3. The corresponding vibration transmission curve was calculated, as shown in Figure 2b.

**Figure 4:**
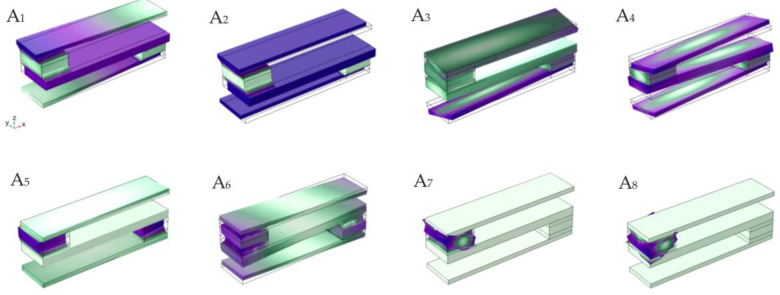
The displacement fields of eigenmodes labeled in Figure 2a.

It can be seen from Figure 2a that the band structure of the S-type oscillator opens two wide band gaps between 57–96 Hz and 252–654 Hz, and it can be seen from Figure 2b that vibration is transmitted in a finite structure, and the attenuation frequency domain of the transmission curve is also very wide, roughly corresponding to the band structure. This is consistent with the conclusion that the band gap frequency band of infinite structure coincides with the attenuation frequency band of finite structure, which has been obtained in previous studies. Such a low-frequency band gap of an S-type local oscillator has a considerable application prospect for realizing low-frequency vibration reduction in ships in a specific frequency range. In order to reveal its band gap characteristics, the displacement fields corresponding to the characteristic modes of critical points in the band structure diagram are extracted, as shown in Figure 4.

In the band structure of the S-shaped local oscillator, for modes A_1_ and A_2_, the displacement field is the longitudinal vibration of the steel plate, while the glass fiber is still. The longitudinal vibration dynamic balance is reached between the positive vibration phase of the upper and lower steel plates and the negative vibration phase of the middle steel plate. For the modes A_3_ and A_4_, the displacement field has a certain degree of torsional vibration. The difference, however, is that, in mode A_3_, the top and bottom plates are subjected to a degree of torsional vibration while the middle plate remains stationary. In mode A_4_, the upper, middle, and lower steel plates are subjected to a certain degree of torsional vibration while the FRP remains stationary. For modes A_5_ and A_6_, the displacement field is mainly the vertical vibration of the composite block along the Z direction, and the steel plate remains stationary. However, in mode A_5_, the composite block is in the state of compression, while in mode A_6_, the composite block is in the state of tension. For modals A_7_ and A_8_, the displacement field is the torsional vibration of the composite block. However, the difference is that in mode A_7_, the displacement field is clockwise torsion, while in mode A_8_, the displacement field is counterclockwise torsion. As a result of these mode coupling effects, two band gaps are opened with frequency ranges of 57–96 Hz and 252–654 Hz. Calculation of the vibration transmission curve shows that the attenuation band is essentially the same as the vibration band gap range.

### 3.2. Band Structure, Eigenmode, and Transmission Power Spectrum of Phononic Crystal Sandwich Plates

**Figure 5:**
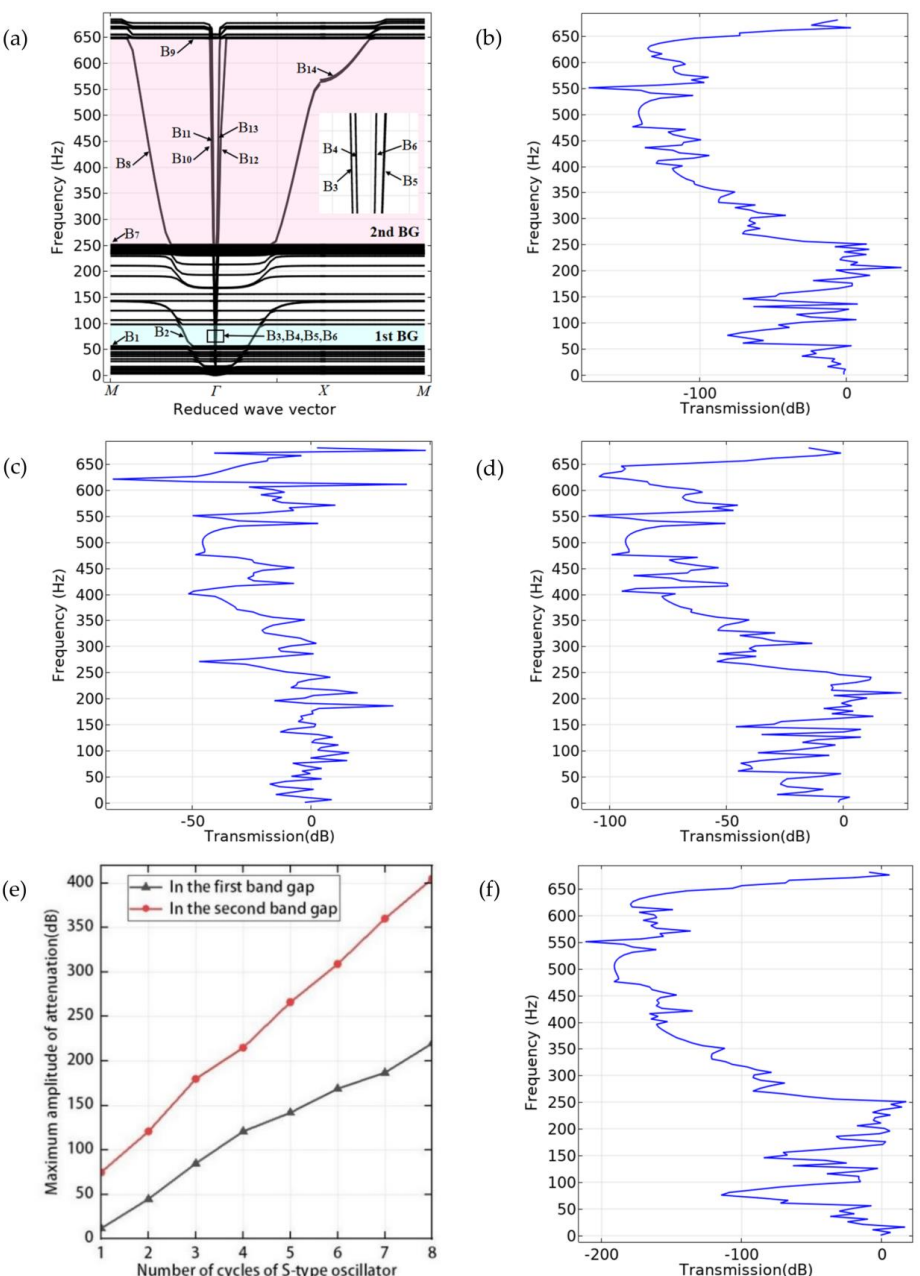
(**a**) Band structure of phononic crystal sandwich plate. (**b**) Transmission power spectrum of sandwich plate with 3 S-type oscillators. (**c**) Transmission power spectrum of sandwich plate with 1 S-type oscillator. (**d**) Transmission power spectrum of sandwich plate with 2 S-type oscillators. (**e**) Relationship between the number of S-type oscillators and attenuation amplitude. (**f**) Transmission power spectrum of sandwich plate with 4 S-type oscillators.

**Figure 6:**
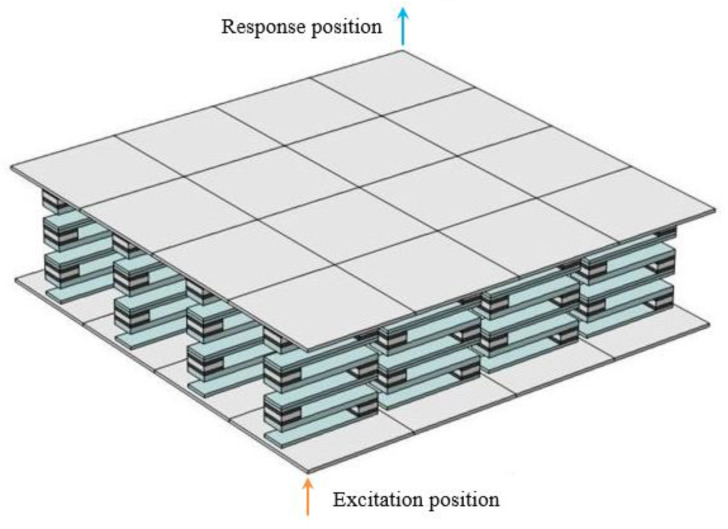
Structure model of phononic crystal sandwich plate composed of 16 cells arranged periodically.

The sandwich plate structure of phononic crystal can be formed by taking three vibrators in a vertical periodic arrangement and attaching an upper plate and a lower plate above and below them. The crystal cell band structure of the plate structure is shown in Figure 5a. The parameters used in the calculation are shown in Table 1 and Table 2. Figure 6 shows the positions of the excitation point and response point, and the vibration transmission power spectrum obtained is shown in Figure 5b.

**Figure 7:**
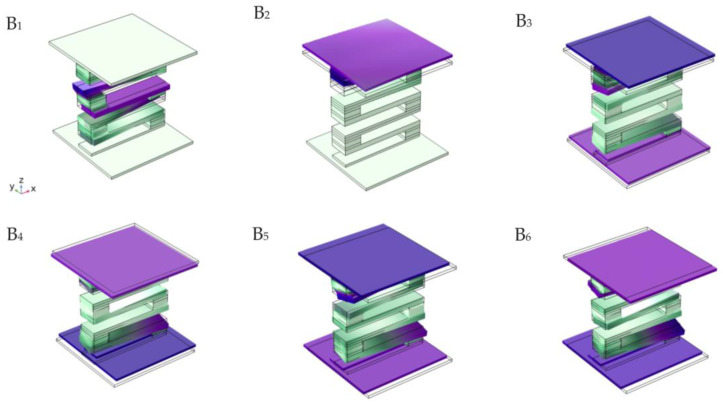
The displacement fields of eigenmodes labeled within the first band gap in Figure 5a.

**Figure 8:**
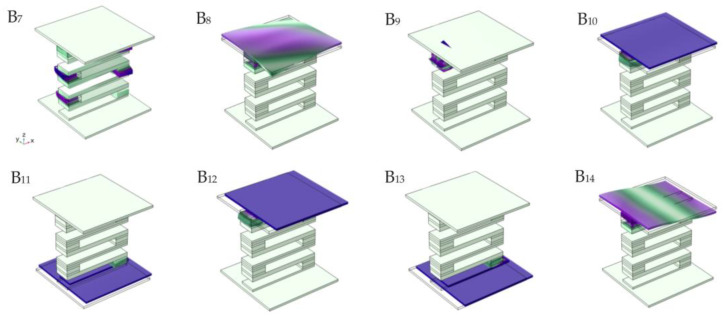
The displacement fields of eigenmodes labeled within the second band gap in Figure 5a.

Figure 5c,d,f show the flexural vibration transmission power spectrum of the 1-period, 2-period, and 4-period S-type oscillator with a sandwich phononic crystal structure, respectively. Figure 5e shows the relationship curve between the number of periods of the S-type oscillator and the maximum attenuation amplitude. It can be seen from the figure that the flexural vibration attenuation of a phononic crystal sandwich plate structure with a periodic S-type oscillator is very small. With the increase in the period number of the S-type oscillators, the attenuation of the first band gap and the second band gap frequency becomes larger and larger, which can be intuitively seen from the relationship curve between the period number of the S-type oscillators and the maximum attenuation amplitude. This is because, when the frequency is within the band gap of the S-type oscillator, the flexural wave cannot travel through the S-type oscillator. The S-type oscillator is periodically attached between the upper plate and the lower plate, which is equivalent to embedding a one-dimensional phononic crystal into the new structure, forming a new phononic crystal structure, and greatly improving the vibration isolation effect of flexural vibration. Theoretically, the greater the number of cycles, the better the vibration reduction effect. However, due to the limitation of structure size and the requirement of being lightweight, it is necessary to use a small period to meet the expected vibration reduction effect. It can be seen from Figure 5a that the band structure does not have an obvious complete band gap, but its corresponding vibration transmission power spectrum has a very wide attenuation frequency domain, and the range roughly corresponds to the attenuation frequency domain of the S-type oscillator. In order to discover the band gap characteristics and reveal the phenomena displayed, displacement fields are created in the corresponding attenuation frequency domain. The displacement fields corresponding to the modes in the first band gap are shown in Figure 7, and those corresponding to the modes in the second band gap are shown in Figure 8.

Figure 7 shows the displacement field corresponding to the characteristic modes marked in the first gap in Figure 5a. Mode B_1_ is the critical point in the first gap, and the vibration energy is concentrated in the S-type vibrator. The displacement field is the longitudinal vibration of the steel plate in the vibrator, and the top and bottom plates remain static. The phase of the middle plate moving in the negative direction of the y axis and that of the top and bottom plates moving in the positive direction of the Y axis reach the dynamic balance of longitudinal vibration, which is just similar to mode A_1_. The displacement field corresponding to mode B_2_ is the vertical vibration of the upper plate, while the lower plate and the sandwich of the S-shaped vibrator remain basically static. For the modes B_3_–B_6_, the corresponding displacement fields are the longitudinal vibration of the top and bottom plates, and the S-shaped interlayer is basically static. In modes B_3_ and B_5_, the top and bottom plates reach the longitudinal vibration dynamic equilibrium with the same phase, which is called the symmetric longitudinal vibration mode. In modes B_4_ and B_6_, the top and bottom plates reach longitudinal vibration dynamic equilibrium with opposite phases, which is called the antisymmetric longitudinal vibration mode. In addition, it can be seen from Figure 7 that the vibration phase of the upper plate in mode B_3_ is opposite to that in mode B_4_. Similarly, the vibration phase of the upper plate in mode B_5_ is opposite to that of the upper plate in symmetric longitudinal vibration mode B_6_. Therefore, when the vibration modes B_3_–B_6_ are superimposed, the vibration of the upper plate is weakened, which is the reason why there is a very wide attenuation domain in the vibration transmission curve when the excitation points and response points are placed on the top and bottom plates, respectively, as shown in Figure 5b.

Except for mode B_1_, which is the critical point in the first gap, the other modes are all within the frequency domain of the first gap. In addition, the displacement field corresponding to the mode extracted from the first gap of the phononic crystal plate structure demonstrates that in the critical point mode B_1_ of the first gap, the longitudinal vibration of the steel plate in the S-type oscillator exactly corresponds to the displacement field of the critical point mode A_1_ of the S-type oscillator in the first gap. For modes B_2_–B_6_ within the range of the first gap, the displacement fields are vibrations of the top and bottom plates, and the sandwich of the S-shaped vibrator basically remains static. In other words, only the displacement field corresponding to mode B_1_ has vibration energy concentrated in the sandwich of the S-type vibrator, while the displacement field corresponding to other modes has vibration energy concentrated on the top and bottom plates, and the first gap of the phononic crystal plate structure and the first gap of the S-type vibrator correspond to the same frequency band. Therefore, from the modal point of view, the opening of the first gap in the phononic plate structure is the result of the coupling of these modes. From the point of view of the structure, the phononic crystal plate structure uses the first gap of the S-type oscillator to open the first gap of 57–96 Hz.

Modes B_7_ and B_9_ are the critical points in the second band gap, whose displacement field is that the top and bottom plates remain static, and the S-shaped oscillator vibrates. Mode B_7_ is a coupling mode in which two compound blocks move along the positive direction of the y axis and two compound blocks move along the negative direction of the Y axis in the S-shaped oscillator, thus achieving dynamic equilibrium. Mode B_9_ is the counterclockwise rotation of a composite block in the S-shaped oscillator, which corresponds exactly to mode A_8_. For modes B_8_ and B_14_, just in the range of the second band gap, the displacement field is such that the top plate vibrates while the bottom plate and the S-shaped oscillator remain essentially static. The difference is that the displacement field of mode B_8_ is the vertical vibration of the upper plate and the uppermost S-type vibrator, while the displacement field of mode B_14_ is the clockwise torsional vibration of the upper plate and the vertical vibration of the uppermost S-type vibrator. In modes B_10_–B_13_, the displacement field is the longitudinal vibration of the upper plate or the lower plate along the plate direction, while the S-shaped oscillator and the other plate remain basically static. It can be seen from Figure 8 that in the range of the second band gap, no matter how the lower (upper) laminates vibrate in the displacement field in these modes, the upper (lower) laminates and the S-shaped vibrators remain static, indicating that the vibration of one plate will not be transmitted to another plate and that the structure has good vibration isolation performance in the corresponding frequency domain. This is because in the attenuation frequency domain, the frequency falls within the band gap band of the intermediate oscillator, and the S-type oscillator in the center of the structure plays a key role in isolation and excitation. The vibration of one plate cannot be transmitted to another plate through the S-type oscillator in the middle of the plate structure, so the second band gap is opened with a frequency range of 252–654 Hz.

**Figure 9:**
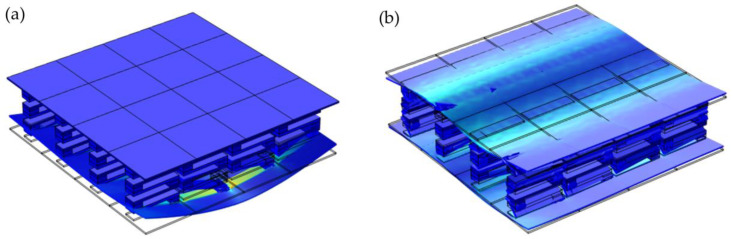
Vibration modes of finite phononic crystal plates with frequencies in different ranges. (**a**) The frequency range of attenuation (*f* = 80 Hz). (**b**) The range of frequencies that are not decaying (*f* = 126 Hz).

In order to further illustrate the attenuation characteristics, the vibration modes of the sandwich plate structure with a finite phononic crystal whose frequency falls in the attenuation domain are given, as shown in Figure 9a. Here, the model used in the calculation is shown in Figure 6. The excitation point applies the displacement excitation in the z direction, and the frequency point is selected as *f* = 80 Hz. It can be seen from Figure 9a that when all the excitation is applied to the lower plate, the vibration cannot be transmitted to the upper plate through the S-shaped oscillator. In general, when the vibration source and response position are on the opposite side of the sandwich structure, it can be considered that two band gaps of flexural vibration attenuation in the corresponding finite structure are opened. The mode diagram of the finite two-layer plate structure, whose frequency falls in the non-attenuation domain, is shown in Figure 9b. The frequency point is selected as *f* = 126 Hz. It can be seen that when all the excitation is applied to the lower plate, the vibration can be transmitted to the upper plate to a certain extent.

### 3.3. Experimental Validation

**Figure 10:**
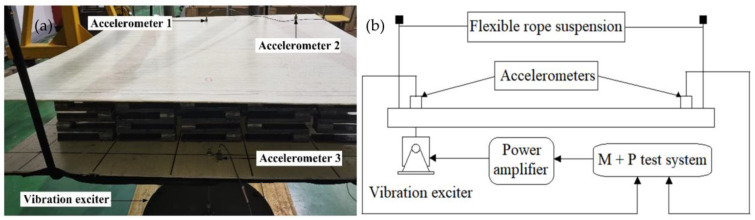
(**a**) The experimental setup. (**b**) The experimental measurement principle.

In an attempt to further verify the correctness of the numerical calculation, a finite period sandwich plate structure consisting of five cells in the x direction and five cells in the y direction was tested. The experimental test system consists of the M + P test system, a power amplifier, a computer for installing a control software, analysis software, three accelerometers, and a vibration exciter. The experimental measurement system is shown in Figure 10a, and the experimental measurement principle is shown in Figure 10b.

During the experimental test, the sandwich plate structure was suspended by a flexible rope to achieve free boundary conditions. The vibration exciter below the plate structure is connected to the power amplifier, and the signal generator sends a continuous sinusoidal signal with a steady amplitude to the power amplifier. Three accelerometers were installed to measure the input and output acceleration frequency responses, and the transmission spectrum can be calculated by the following equation:

(4) $$T=20\mathrm{lg}\frac{\left|a_{out}\right|}{\left|a_{in}\right|}$$

In order to facilitate the experimental operation, the following geometric parameters are designed. The material parameters of the sample were measured by a quasi-static tensile test. The geometric dimensions and material parameters of the numerical simulation were consistent with those of the experimental samples, as shown in Table 3 and Table 4. The material parameters of FRP are: density m = 2075 kg/m^3^; Young’s modulus E_1_ = E_2_ = 22.6 GPa, E_3_ = 13.5 GPa; Poisson’s ratio v_1_ = v_2_ = 0.22, v_3_ = 0.13.

**Figure 11:**
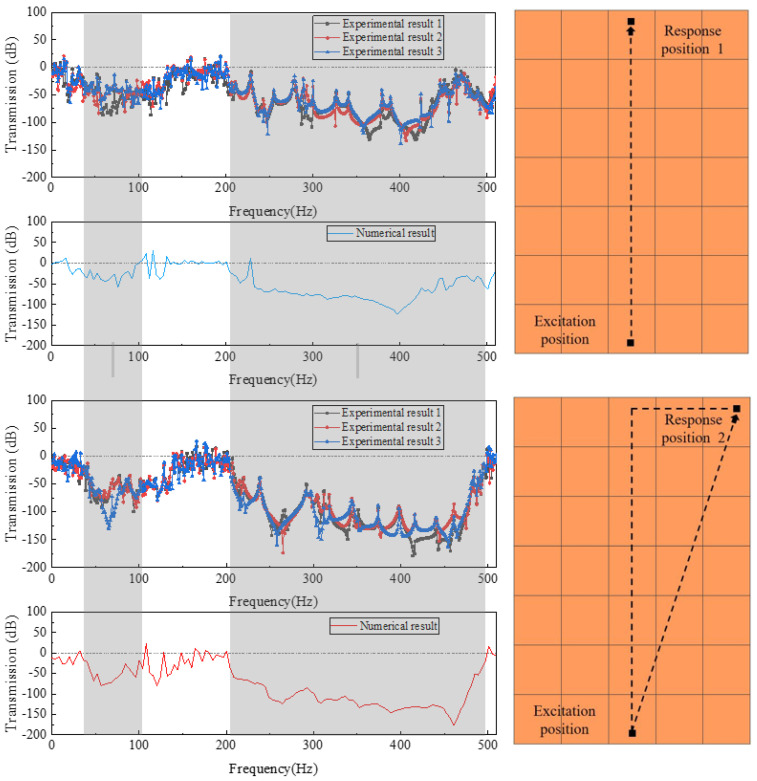
Experimental validation of flexural vibration frequency responses.

The experimental setup is shown in Figure 10a. The vibration transmission characteristics obtained from the experiment are compared with the numerical calculation results. As can be seen from Figure 11, the flexural vibration frequency response characteristic curve of the phononic crystal sandwich plate structure has obvious attenuation, showing excellent low-frequency vibration isolation performance. The experimental results showed good agreement with the numerical results, which demonstrated the accuracy and reliability of the numerical method.

## 4. Application of Phononic Crystal Sandwich Plate in Ship Vibration Reduction

**Figure 12:**
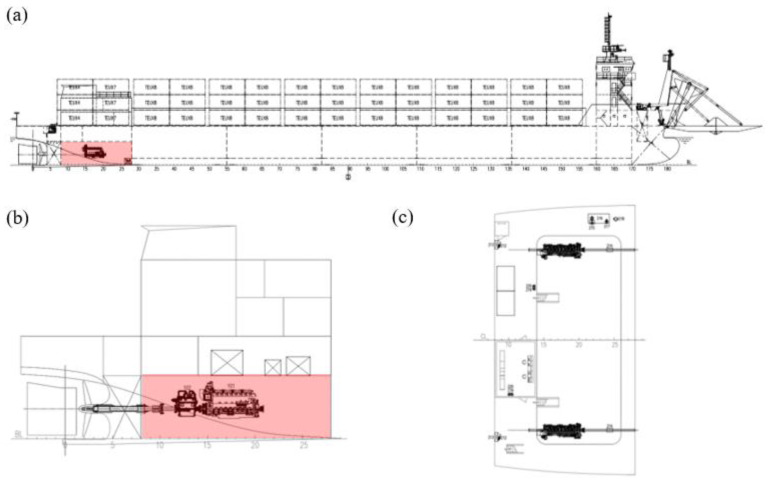
(**a**) A 113-m-offshore deck cargo ship. (**b**) Power cabin of ship. (**c**) Platform of power cabin.

Based on the above research, we explore the practical application of vibration control in marine power systems. Taking the power cabin of a 113-m-offshore deck cargo ship as an example, this paper calculates the vibration isolation performance of the bulkhead and vibration isolation platform, respectively, when the phononic crystal sandwich plate structure is applied in the ship’s power system (it has been marked red in the Figure 12). Considering the size of units and computation, this paper simplified the model of the actual structure and converted the phononic crystal sandwich plate structure cell into the real ship proportion. The geometric parameters are shown in Table 5.

### 4.1. Vibration Characteristics of the Ship Power System Steerage Bulkhead

**Figure 13:**
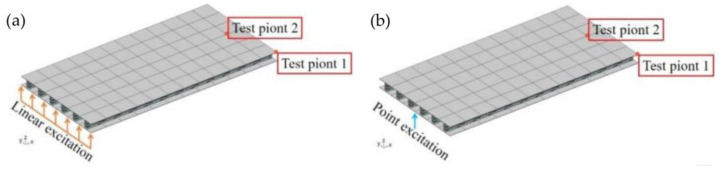
Schematic diagram of a phononic crystal sandwich plate structure applied to vibration control of the ship’s power system bulkhead. (**a**) Under a linear excitation. (**b**) Under a point excitation.

In order to prove the vibration isolation performance of a phononic crystal sandwich plate structure in the ship power system on the actual ship scale, the engine room bulkhead is simplified into a plate with a length of 5.28 m, a width of 2.64 m, and a thickness of 23.2 cm, as shown in Figure 13. It can be observed that the simplified plate is composed of 6 × 12 phononic crystal sandwich plate cells. A linear or point excitation source is applied to one side of the sandwich plate to simulate the excitation of the ship’s power system.

**Figure 14:**
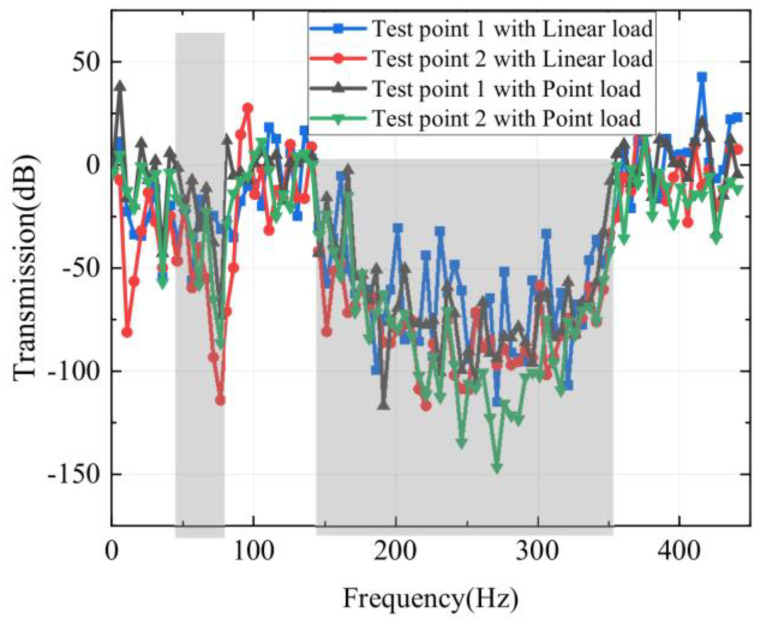
Flexural vibration frequency responses of real ship-scale phononic crystal sandwich plate structure.

Figure 14 calculates the flexural vibration frequency response of the sandwich plate structures at the actual ship scale. The test position is shown in Figure 13, and the flexural vibration frequency responses of test points 1 and 2 are calculated, respectively, under linear excitation and point excitation. It can be found that there is an attenuation region (The gray area) of flexural vibration from 45–78 Hz to 145–355 Hz in the frequency response, which indicates that the sandwich plate cell structure of a real ship size can achieve low-frequency vibration isolation. Regardless of linear excitation or point excitation, the flexural vibration frequency response characteristic curves of test point 1 and test point 2 are basically the same. It can be concluded that the real-size sandwich plate structure can play an important role in the application of low-frequency vibration and noise reduction technology on ships.

### 4.2. Vibration Isolation Characteristics of a Vibration Isolation Platform

**Figure 15:**
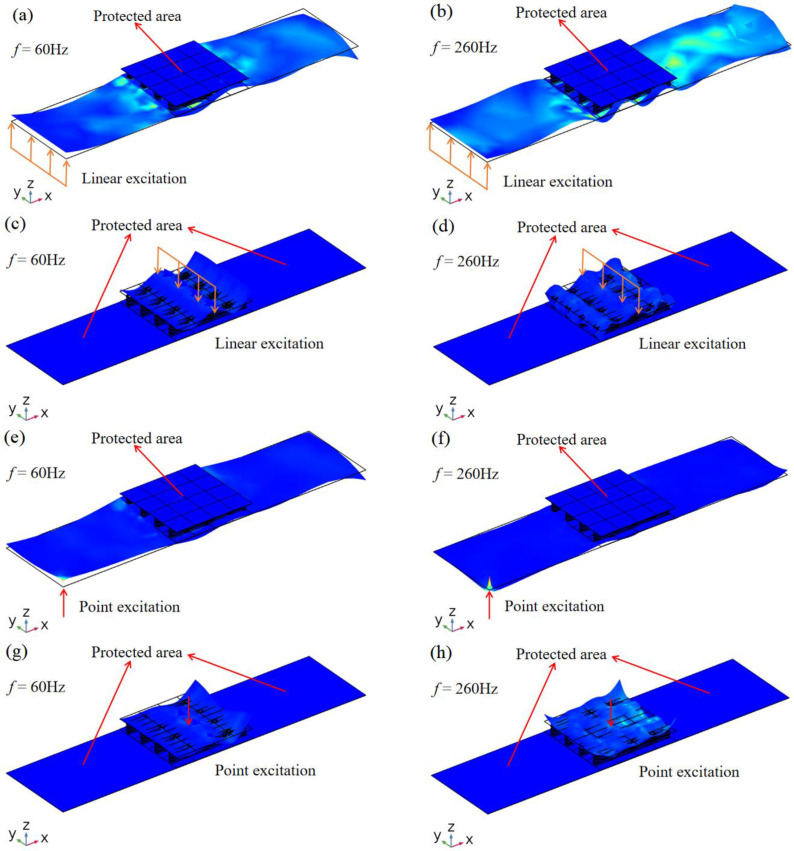
Displacement field of vibration isolation platform under different excitation. (**a**) When f = 60 Hz, under linear excitation at the left. (**b**) When *f* = 260 Hz, under linear excitation at the left. (**c**) When *f* = 60 Hz, under linear excitation in the middle section. (**d**) When *f* = 260 Hz, under linear excitation in the middle section. (**e**) When f = 60 Hz, under point excitation at the left. (**f**) When *f* = 260Hz, under point excitation at the left. (**g**) When *f* = 60 Hz, under point excitation in the middle section. (**h**) When *f* = 260 Hz, under point excitation in the middle section.

Based on the phononic crystal sandwich plate structure shown in Figure 13, a vibration isolation platform composed of phononic crystals mounted on the host board is designed. As shown in Figure 15, a phononic crystal sandwich structure (4 × 4 cycles) is arranged on a steel plate. The total length and thickness of the motherboard are 7.04 m and 6 mm, respectively. Similar to the simulation in Figure 13, the performance of a vibration isolation platform under a linear excitation source or a point excitation source is shown, respectively. It can be seen that whether the excitation source is linear or a point, as long as it is in the first or second band gap region, the elastic wave can only propagate on one side of the sandwich structure, but not on the other side. If the excitation source is applied to the lower host plate, the elastic wave will propagate in the host plate, but will not affect the protected area on the upper side of the phononic crystal sandwich structure. When the excitation source is applied to the upper side of the phononic crystal sandwich structure, the elastic wave cannot propagate to the protected region of the lower side. The results not only verify the calculation results in Figure 14, but also show that the vibration isolation platform can achieve a good vibration isolation effect within the band gap range.

## 5. Conclusions

A phononic crystal plate structure was proposed to solve the problem of low-frequency vibration control of ship power systems. The band structure, displacement field corresponding to the characteristic modes, and vibration transmission curve of the corresponding finite period two-layer plate structure were calculated with a finite element method, and the band gap characteristics of the structure were studied. For the plate structure, the author designed the S-type oscillator structure. First, the research shows that the S-type oscillator has the characteristics of a low-frequency band gap. It was arranged periodically along the z direction and placed between the upper and lower panels to form a phononic crystal plate cell structure. The cells were arranged periodically along the x and y directions, and finally constitute the phononic crystal plate structure with double periodicity, that is, the one-dimensional periodicity in the z direction of the S-type oscillator and the two-dimensional periodicity in the x and y directions of the sandwich plate. This design can achieve the purpose of widening the band gap or generating a multi-segment band gap, making the control frequency band wider and wider. Moreover, since the S-type oscillator was arranged periodically along the z direction, the transmission of flexural waves can be more fully suppressed, and the low-frequency flexural vibrations can be isolated. The accuracy and reliability of the numerical method were verified by the experimental measurement of the transmission spectra of flexural vibration. With these advantages, this phononic crystal plate structure can be used to protect precision instruments and electronic equipment on many ships. Finally, the potential application of the proposed method to marine power systems is discussed. The following conclusions can be obtained:

1. By analyzing the band structure and transmission power spectrum, the proposed phononic crystal sandwich structure has significant low-frequency flexural band gap and vibration isolation characteristics. The results of band structure and transmission curve are consistent, and the experimental results are consistent with the numerical results, which proves the accuracy and reliability of the numerical method.
2. The period number of the S-type oscillator also has an important influence on the band gap; the higher the period number, the better the vibration reduction effect. The maximum attenuation amplitudes of the first band gap and the second band gap increase with the increase in the period number of the S-type oscillator.
3. For the formation mechanism of the band gap, from the perspective of mode, the opening of the band gap in the phononic plate structure is the result of modal coupling and the weakening of an elastic wave. From the point of view of the structure, the phononic crystal plate structure uses the band gap of the S-type oscillator to open the band gap of the flexural wave consistent with its band gap.
4. The sandwich plate structure of a real ship has a medium- and low-frequency flexural band gap and vibration suppression region in the frequency range of 45–78 Hz and 145–355 Hz, indicating that the proposed ship vibration isolation method has a good application prospect in medium- and low-frequency vibration control of the marine power system.
5. The vibration isolation platform mounted on a steel plate is studied by numerical simulation. The phononic crystal plate structure has good adaptability and flexibility to various loads, regardless of the linear excitation or point excitation, and it can isolate low-frequency vibration to protect electronic equipment and precision instruments.

## Figures and Tables

**Table 1:** Materials’ parameters used in calculations.

Material	Density (kg/m^3^)	Young’s Modulus (GPa)	Poisson’s Ratio
Steel	7780	210.6	0.300
SR	1300	11.75 × 10^−5^	0.469

**Table 2:** Geometric parameters used in calculations (unit/mm).

Parameters	*a*	*e*	*h*_1_	*h*_2_	*h*_3_	*h*_4_	*h*	*l*	*m*
Dimension (mm)	110	3	3	3	6	6	36	100	24

**Table 3:** Materials’ parameters used in the experiment.

Material	Density (kg/m^3^)	Young’s Modulus (GPa)	Poisson’s Ratio
Steel	7812	212.4	0.270
SR	1303	17.75 × 10^−5^	0.471

**Table 4:** Geometric parameters used in the experiment (unit/mm).

Parameters	*a*	*e*	*h*_1_	*h*_2_	*h*_3_	*h*_4_	*h*	*l*	*m*
Dimension (mm)	150	4	4	4	8	8	48	135	32

**Table 5:** Dimensions of the sandwich plate structure at the real ship scale (unit/mm).

Parameters	*a*	*e*	*h*_1_	*h*_2_	*h*_3_	*h*_4_	*h*	*l*	*m*
Dimension (mm)	440	6	6	6	12	12	72	400	96
